# Transmembrane Recognition of the Semaphorin Co-Receptors Neuropilin 1 and Plexin A1: Coarse-Grained Simulations

**DOI:** 10.1371/journal.pone.0097779

**Published:** 2014-05-23

**Authors:** Samia Aci-Sèche, Paul Sawma, Pierre Hubert, James N. Sturgis, Dominique Bagnard, Laurent Jacob, Monique Genest, Norbert Garnier

**Affiliations:** 1 Centre de Biophysique Moléculaire, Centre National de la Recherche Scientifique UPR, Université d’Orléans, Orléans, France; 2 Laboratoire d’Ingénierie des Systèmes Macromoléculaires UMR, Université d’Aix-Marseille, Marseille, France; 3 Institut National de la Santé et de la Recherche Médicale, Labex Medalis, Université de Strasbourg, Strasbourg, France; Instituto de Tecnologica Química e Biológica, UNL, Portugal

## Abstract

The cancer associated class 3 semaphorins require direct binding to neuropilins and association to plexins to trigger cell signaling. Here, we address the role of the transmembrane domains of neuropilin 1 and plexin A1 for the dimerization of the two receptors by characterizing the assembly in lipid bilayers using coarse-grained molecular dynamics simulations. From experimental evidence using a two-hybrid system showing the biochemical association of the two receptors transmembrane domains, we performed molecular simulations in DOPC and POPC demonstrating spontaneously assembly to form homodimers and heterodimers with a very high propensity for right-handed packing of the helices. Inversely, left-handed packing was observed with a very low propensity. This mode of packing was observed uniquely when the plexin A1 transmembrane domain was involved in association. Potential of mean force calculations were used to predict a hierarchy of self-association for the monomers: the two neuropilin 1 transmembrane domains strongly associated, neuropilin 1 and plexin A1 transmembrane domains associated less and the two plexin A1 transmembrane domains weakly but significantly associated. We demonstrated that homodimerization and heterodimerization are driven by GxxxG motifs, and that the sequence context modulates the packing mode of the plexin A1 transmembrane domains. This work presents major advances towards our understanding of membrane signaling platforms assembly through membrane domains and provides exquisite information for the design of antagonist drugs defining a novel class of therapeutic agents.

## Introduction

Most of cell activities are controlled by membrane receptors, which in response to extracellular ligands trigger intracellular signaling reactions. A hallmark of these signaling events is the reversible formation of multicomponent complexes, which associate membrane and cytosolic proteins through specialized protein binding modules [1]. The first step in the assembly of these multiprotein complexes is usually the dimerization/oligomerization of the receptors themselves, or structural rearrangements of preformed oligomers. Examples of such receptor complexes include receptor tyrosine kinases such as the ErbB family or Ephrin receptors [2], the T-cell receptor complex which is part of the immunological synapse [3], integrins [4], G-protein coupled receptors [5] among others.

In many cases, the assembly of these signaling platforms involves interactions between the transmembrane domains of the receptors. Different studies have reported the functional importance of intramembrane interactions for membrane protein signaling [6]–[12]. The role of the transmembrane (TM) domains depends on their propensity to associate and on the existence of specific dimerization motifs in their sequence. The core of the dimerization motif of TM domains is often a GxxxG sequence (where G is glycine or another small residue, and x represents any amino-acid). This motif was first recognized in the case of glycophorin A and was since found in many other examples of interacting TM helices [13]–[18]. A detailed view on how such TM interactions are involved in the mechanism of activation of a receptor has very recently been provided for the EGF receptor, a receptor tyrosine kinase. Using NMR, molecular dynamics simulations and biochemical assays, J. Kuriyan’s group has shown that ligand binding causes a rearrangement of TM helices in an EGFR dimer, so that the juxtamembrane and kinase domains can form the activated asymmetric dimer [19], [20].

Semaphorins form a large family of membrane-associated and secreted proteins playing many roles in a variety of cellular processes. They are bifunctional signaling molecules capable of growth promoting or growth inhibitory effects [21]. The diversity of functions encoded by semaphorins is related to the formation of specific receptor complexes. Together with their receptors, the neuropilins and the plexins, semaphorins are the constituents of a complex regulatory system responsible for axon guidance during the development of the central nervous system [22], [23]. Neuropilins are also involved in vascular development and tumorigenesis [24]. The secreted semaphorins of subclass 3 (Sema3A-Sema3G) bind directly to neuropilins with different affinities for the two members of the family, neuropilin 1 and neuropilin 2 [25], [26] but do not bind directly to plexins [27]–[29]. The functional receptor for secreted semaphorins is a complex including neuropilins and plexins. Thus, neuropilins form signaling complexes by associating with type A plexins to activate downstream signal transduction cascades. In these complexes, neuropilins are not able to transduce semaphorin signals owing to their short intracellular domains but act as binding receptors while plexins act as signal-transducing components [30].

Various studies have revealed that the extracellular domain of plexin A1 (PLXA1) interacts with neuropilin 1 (NRP1), allowing a Sema3 signal directly to the cytoplasm via the intracellular domain of plexin A1 [31]–[33]. It appears that the sema domain at the extracellular region of semaphorins and plexins is a common structural element able to mediate a large variety of protein–protein interactions, particularly semaphorin-plexin binding and semaphorin-neuropilin binding. The sema domain of plexins has been suggested to mediate the formation of ligand independent homodimers. Moreover, with their ligands, homodimers represent the active forms of Sema3A and likely of all semaphorins [34], [35]. Structural studies have partially elucidated the binding mode between plexins and semaphorins [36]–[40]. Nevertheless, the data do not clarify the mechanism by which semaphorin-plexin A1 interactions, together with neuropilin binding, lead to the activation of the cytoplasmic region of plexins.

Several hypotheses have been proposed [32], [40]–[42]. Recent findings clearly show that semaphorin dimers are needed for signaling [36], [39] and that the core of the mechanism of semaphorin-mediated plexin dimerization is central to the Sema3 function. The requirement of neuropilin as co-receptor to stabilize this complex is believed to induce neuropilin dimerization/oligomerization or receptor clustering and may possibly induce clusters of the plexin intracellular region [36], [39], [43], [44]. The architecture of the Sema3-plexin A complexes and the role of the extracellular domains in dimerization have been revealed by crystal structures [36], [38], [39]. Recently, the central role of the extracellular domain of neuropilins in Sema3-plexin A signaling has been demonstrated [43]. Thus, the extracellular domains are required for formation of the complex, but very probably, other binding sites are necessary to trigger the function of the semaphorin-neuropilin 1-plexin A1 complex. Identifying these sites represents an important issue in understanding the dynamic behavior of these receptors.

One such site could be the TM domain of neuropilin 1 (NRP1 TM) which contains a conserved double GxxxG motif. Mutations of this motif confirmed its biological importance for Sema3A signaling [45]. Moreover, this same study showed that this domain exhibits a strong dimerization capacity. Furthermore, the TM domain of plexin A1 (PLXA1 TM) contains six Gly residues constituting a GxGGGGG (or GxG5) motif. This Gly rich motif could thus be expected to be involved in helix-helix interactions. Unfortunately, experimental data on the role of this TM domain in association processes are not available to date. These motifs have been widely described as mediators for TM dimerization and oligomerization. For neuropilin 1, the ability of the TM domain to form dimers was initially demonstrated by a two-hybrid assay performed in bacterial membranes and by Föster Resonance Energy Transfer (FRET) analysis [45]. A major functional role for these TM interactions was evidenced by the potent antagonistic effect of a synthetic peptide mimicking the NRP1 TM domain on neuropilin signaling, both *in vitro* and *in vivo* [21], [46]. Mutations of the GxxxG motifs rendered the peptide and the receptor completely inactive, thus confirming their functional importance. Moreover peptides targeting and inhibiting the TM of NRP1 turn out to be very potent anti-cancer drug [46] contributing to the identification of NRP1 as a major therapeutic target in cancer [47].

Insights into activation mechanisms require information about the conformational dynamics of TM interactions, not yet available experimentally. Consequently, to explore the relationship between semaphorin binding proteins, we sought to characterize the molecular interactions between these TM domains and determine how the putative interaction motifs contribute to the stabilization/destabilization of the associated TM complexes.

In this study, we first used a two-hybrid system to demonstrate homotypic and heterotypic interactions of the TM sequences of NRP1 and PLXA1. Then, we employed coarse-grained molecular dynamics (CG-MD) simulations to explore the self-assembly of the NRP1 and PLXA1 TM domains within lipid bilayers [48]–[50] composed of pure DOPC (1,2-di-oleoyl-sn-glycero-3-phosphocholine) and pure POPC (palmitoyl-2-oleoyl-sn-glycero-3-phosphocholine). The success of these techniques in understanding the behavior of lipid membranes and membrane proteins [51] opens the route to investigating the association properties of these domains in homotypic and heterotypic forms. The potential of mean force (PMF) has been calculated and differences in free energies of association are compared. To the best of our knowledge, this work is the first devoted to the investigation of the association of the TM domains of the semaphorin co-receptors in membrane bilayers using CG-MD simulations.

## Results

### Dimerization Propensity of NRP1 and PLXA1 TM Sequences in BACTH

**Figure 1:**
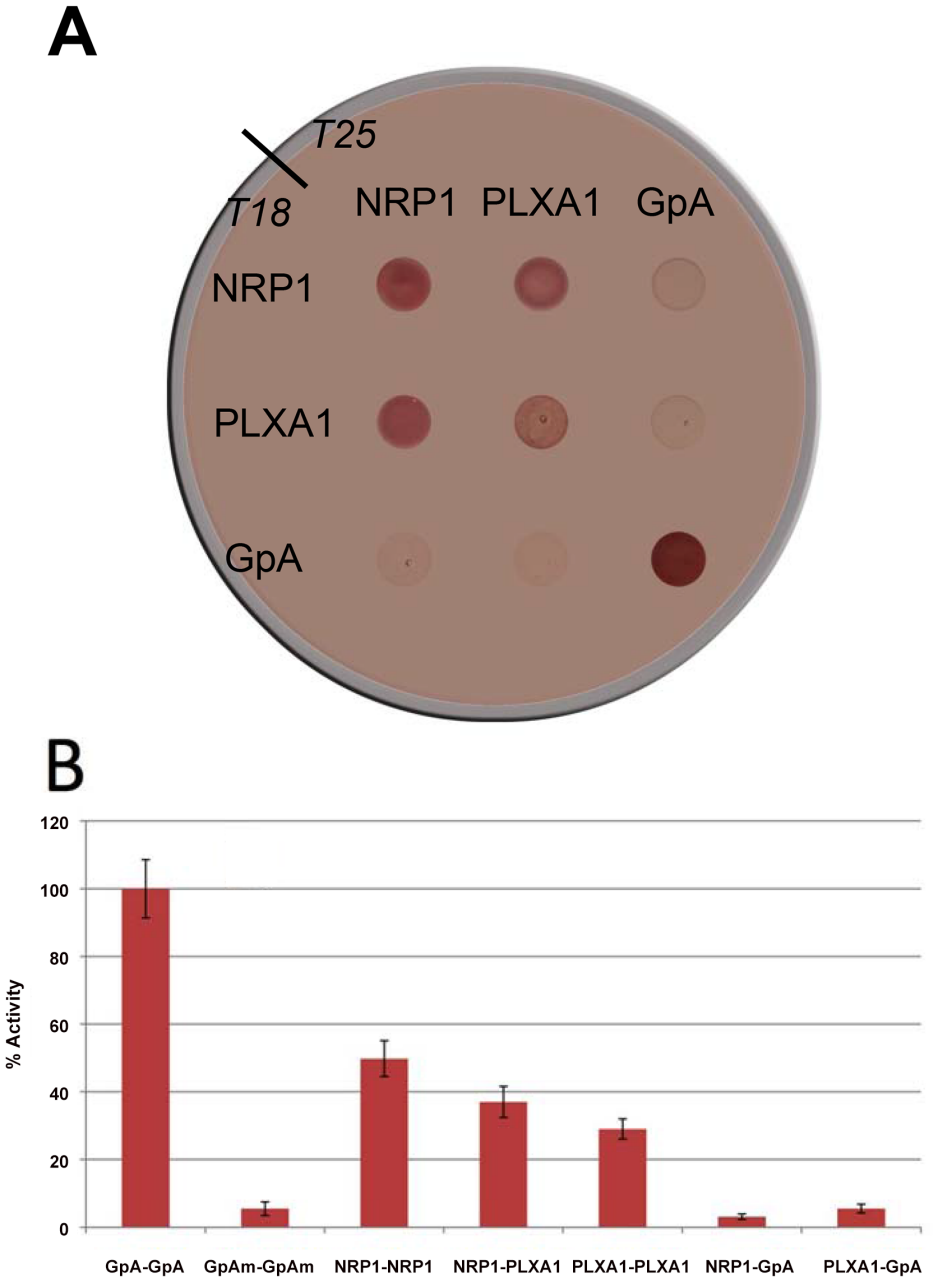
Bacterial adenyl cyclase two-hybrid (BACTH) assay of NRP1 and PLXA1 TM sequences. *E. coli* BTH101 host cells were cotransformed with a pair of plasmids encoding adenyl cyclase domains T18 and T25 fused to the indicated TM sequences. Cells were directly plated on MacConkey indicator plates (panel A). The fusions with the TM domain of glycophorin A (GpA) are a positive control for homotypic interactions. Red coloration of the colonies indicates a cya+ phenotype and thus interaction of the two TM domains. In panel B, the efficiencies of functional complementation between the indicated TM domains were quantified by measuring β-galactosidase activities in *E. coli* BTH101 harboring the corresponding plasmids as described in Materials and Methods. Values are normalized relatively to GpA taken as 100%.

To assess first the propensities of the TM domains of NRP1 and PLXA1, we employed a bacterial adenylate cyclase two-hybrid assay (BACTH). We used the TM sequence of human Glycophorin A (GpA) as both a positive control for homotypic interactions and a negative control for heterotypic ones. Figure 1 depicts the results of BACTH functional complementation between the TM domains of GpA, NRP1 and PLXA1 (Table 1) determined by growing transformed cells on an indicator plate (panel A) and by measuring β-galactosidase activity (panel B). Both tests indicate clearly that NRP1 and PLXA1 TM domains can self-associate, but also form heterodimers. Expression levels of constructs was checked by Western-blotting and found to be similar for all proteins (Sawma et al., manuscript submitted). The interactions order is as follows: NRP1-NRP1> NRP1-PLXA1>PLXA1-PLXA1. The NRP1-NRP1 interaction propensity is about 50% of that of GpA-GpA, and specificity of the assay is shown by the absence of interactions of the GpA sequence with the other two.

**Table 1:** TM domain sequences of human Neuropilin-1 (NRP1) and Plexin-A1 (PLXA1), protein names, identifiers, entry names taken from the Uniprot database (http://www.uniprot.org/) and TM domain sequences used in CG-MD simulations.

TM domain	Sequence	Protein residues
**Neuropilin-1**	ILITIIAMSAL**GVLLGAVCG**VVL	857–879 (23 aa)
(O14786, NRP1_HUMAN)		
NRP1 TM domain (MD)	^1^TLDPILITIIAMSAL**GVLLGAVCG**VVLY^28^	853–880 (28 aa)
**Plexin-A1**	AIV**GIGGGGG**LLLLVIVAVLI	1245–1265 (21 aa)
(Q9UIW2, PLXA1_HUMAN)		
PLXA1 TM domain (MD)	^1^LLTLPAIV**GIGGGGG**LLLLVIVAVLIAY^28^	1240–1267 (28 aa)

For the sake of clarity, the TM sequences have been re-numbered 1 to 28 throughout this paper.

### Self-assembly of TM Domains by Molecular Dynamics

All the simulations performed in DOPC and POPC bilayers led to the association of the TM domains after their diffusion within the membrane. The time required for the formation of the two homodimers and the heterodimer was variable, from 1 µs to 10 µs, depending on the simulations. The overall simulations indicate that once the TM domains associate, helix-helix contacts were maintained for the rest of the simulation. The time during which the TM domains remained associated is significant compared to the total time of each simulation. No dissociation event was seen with the exception of only one simulation conducted for the two PLXA1 monomers embedded in a POPC bilayer.

**Figure 2:**
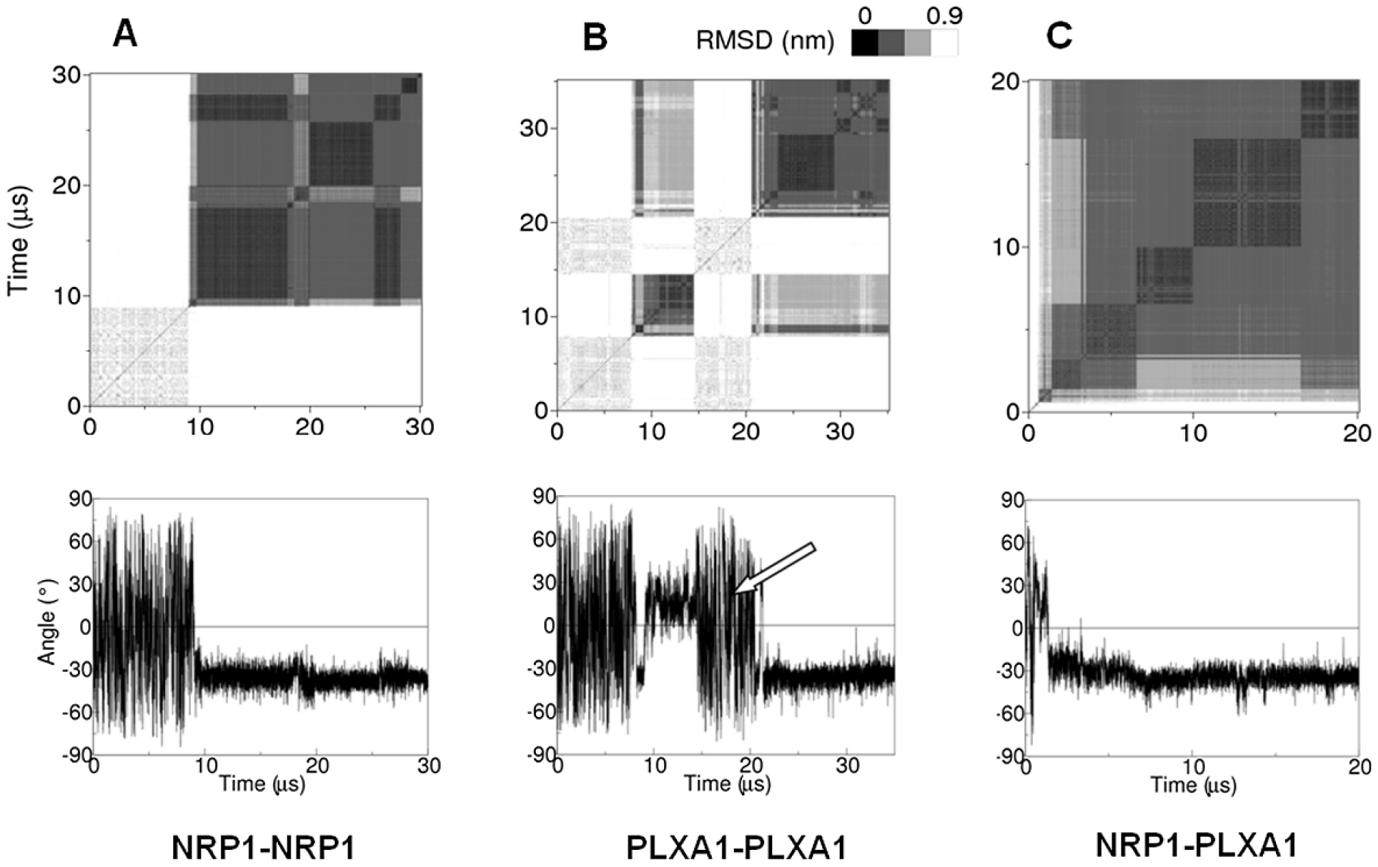
Time-course of the self-assembly of the dimers embedded in a POPC bilayer. RMSD maps and crossing angles illustrate the right-handed association of the TM domains NRP1-NRP1 (A) and NRP1-PLXA1 (C) (negative value of the crossing angle). The two TM domains of PLXA1 (B) associate alternatively in a right-handed or in a left-handed manner along the trajectories. The arrow indicates a transient dissociation event for ∼5 µs seen in one simulation from the left to the right-handed structure.

All RMSD maps and time-courses of the crossing angles were calculated. Three of these maps and their corresponding crossing angles (Figure 2) were chosen to illustrate the formation of each of the three dimers and their behavior during a complete simulation performed in the POPC environment. Self-assembly of the TM domains may happen very early as illustrated here for the NRP1 and PLXA1 monomers (around 1 µs) or later as seen for the two NRP1 monomers (around 10 µs). RMSD maps reveal successive periods for which RMSD values are lower than 0.6 nm, evidencing discrete dimer states very close to each other. These successive periods of low RMSD reflect the dynamics of the two associated monomers within the bilayer.

The RMSD map and the crossing angle representing the time-course of the self-assembly of the PLXA1 TM domains summarize all the characteristics observed for this dimer (Figure 2B). The two periods between 9 µs and 14 µs and between 22 µs and 35 µs reveal two different states, one with the monomers associated in a left-handed manner and the second with the monomers associated in a right-handed manner respectively. These two states are separated by about 5 µs during which the two monomers are fully dissociated. Among all the simulations, this is the only one that reveals a dissociation event.

The complete results of self-simulations of the three dimers embedded in a DOPC bilayer and in a POPC bilayer are given in Table 2. The main feature is that the right-handed association is highly preponderant for the three dimers in the two lipid environments. The crossing angles are similar in the two milieus between −33° and −37° on average with relatively small fluctuations of about 5°–7°. Besides this major property of the three dimers, each has its own particularities.

**Table 2:** Summary of the self-assembly simulations of the dimers embedded in a DOPC bilayer and in a POPC bilayer.

TM Dimers	Lipidbilayer	Total simulationtime (µs)	Total associationtime (µs)	Structure	% associationtime (L, R)	Crossingangle (°)	Inter-helix COMdistance (nm)	Inter-helix contactdistance (nm)
NRP1 homodimer	DOPC	100 (4)	75	L	0			
				R	100	−34 (5)	0.63 (0.12)	0.47 (0.01)
	POPC	110 (4)	93	L	0			
				R	100	−37 (5)	0.54 (0.12)	0.47 (0.01)
PLXA1 homodimer	DOPC	80 (3)	64	L	30	17 (11)	0.87 (0.13)	0.48 (0.03)
				R	70	−33 (7)	0.59 (0.16)	0.48 (0.02)
	POPC	85 (3)	57	L	13	15 (11)	0.89 (0.15)	0.48 (0.07)
				R	87	−35 (6)	0.59 (0.15)	0.48 (0.01)
NRP1-PLXA1 heterodimer	DOPC	118 (6)	83	L	8	24 (7)	0.87 (0.10)	0.48 (0.02)
				R	91	−33 (5)	0.57 (0.12)	0.47 (0.01)
	POPC	120 (6)	101	L	1	20 (8)	0.95 (0.15)	0.48 (0.02)
				R	99	−34 (5)	0.57 (0.12)	0.47 (0.01)

The total simulation time is the sum of the simulation time of the separate MDs. The number in parentheses represents the number of independent simulations. The total association time is the sum of the time of association of the TM domains observed along the separate MDs. The percentage of the association time of the right-handed structures (R) and the left-handed (L) structures is calculated over the total association time.

The values of the crossing angle and the inter-helix distances (COM distances and contact distances) are an average calculated over the last 10 µs of each of the simulations in the cases of the NRP1 homodimer and the heterodimer. For the PLXA1 homodimer the values are an average calculated over the total time of association in right-handed and left-handed interactions.

The very high propensity of the NRP1 monomers to dimerize in right-handed interactions is clearly evidenced in both types of bilayers. This right-handed packing induces short inter-helix contact distances of 0.47 nm with very low fluctuations. Also, inter-helix distances are short, slightly shorter in POPC than in DOPC by 0.1 nm, indicating a weak effect of the nature of the lipids on the TM domains assembly.

The behavior of the PLXA1 TM domains is quite different. First, the two monomers associate spontaneously either in right-handed or in left-handed interactions. Then, for the rest of the simulation, the two monomers cross successively with a positive or a negative angle (or inversely) without dissociation of the monomers, except for the dissociation event mentioned above. For each association mode the mean values of the crossing angle are practically identical in DOPC and POPC in the limit of the fluctuations. These fluctuations are almost twofold greater for a positive angle than for a negative angle, and their magnitude is in the same order as the mean values of the crossing angle. These data reveal the instability of the left-handed structure for this homodimer. The strong dynamics of the monomers crossed in left-handed interactions does not affect their close approach. The inter-helix contact distances are 0.48 nm on average, as found for the right-handed structures in both types of lipids. It should be pointed out that the inter-helix distances depend on the manner in which the two TM domains are crossed, as attested by a difference of about 0.3 nm between the two modes of association.

The propensity of the PLXA1 TM domains to pack in a right-handed or in a left-handed structure is modulated by the nature of the lipids. Left-handed structures are observed for 30% of the total time of association in the DOPC bilayer while in the POPC bilayer they are observed for 13% of this total time. This finding is of interest because it suggests the possibility of equilibrium between the two modes of association.

The self-assembly of the NRP1 and PLXA1 TM domains shows an intermediate behavior between what is observed for the two TM domains NRP1 and PLXA1. The simulations demonstrate a net propensity for the formation of a right-handed structure. Interestingly, left-handed structures are formed but this mode of association is much less common than for the PLXA1 homodimer. These structures are observed for a very low percentage of time in the DOPC bilayer (8%) and for a negligible time in the POPC bilayer (<1%). We note that for this left-handed association, the two NRP1 and PLXA1 monomers are slightly more crossed than the two PLXA1 monomers by about 7° in DOPC and 5° in POPC on average. Inter-helix contact distances are equal on average to those reported for the two homodimers. Inter-helix distances are in the same range as those measured for the PLXA1 homodimer. As mentioned for the PLXA1 homodimer, inter-helix distances in left-handed structures are about 0.4 nm larger than in right-handed structures.

### Helix-helix Interface

**Figure 3:**
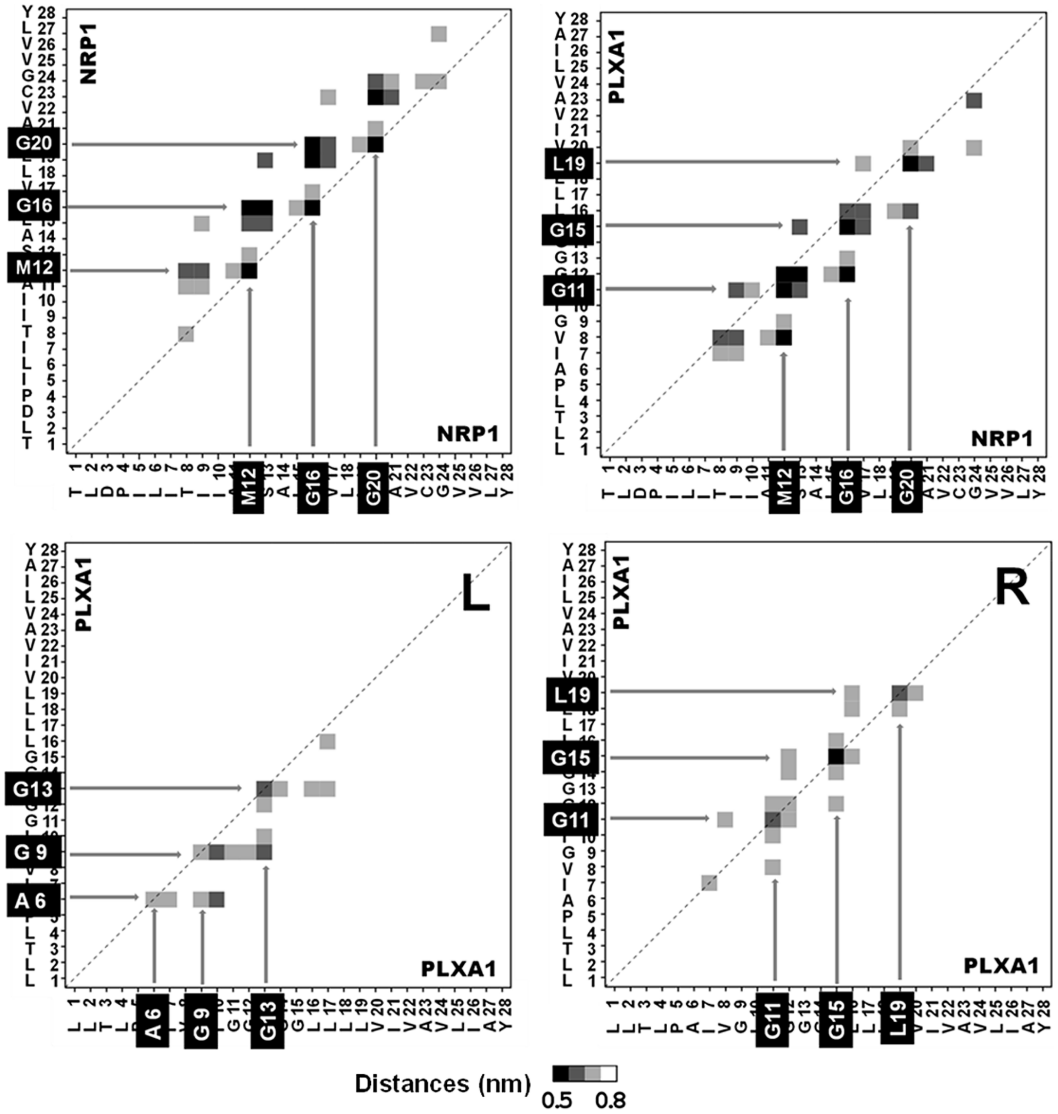
Contact maps of dimers embedded in a POPC bilayer. Top: right-handed structure of the NRP1 homodimer and the NRP1-PLXA1 heterodimer. Bottom: left-handed structure (L) and right-handed structure (R) of the PLXA1 homodimer. Average interfaces highlight the key interfacing residues at distances 0.5 nm<d <0.8 nm. Squares in black, dark gray, and light gray denote the residue pairs at backbone bead distances <0.6 nm, <0.7 nm and <0.8 nm respectively.

**Figure 4:**
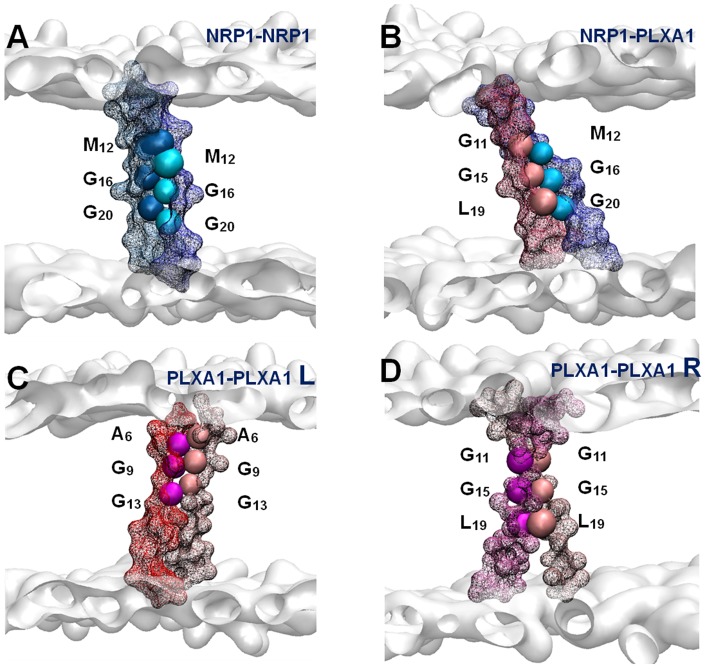
Representation of the dimers in the POPC bilayer. Key interfacing residues depicted on the contact maps (Figure 3) are highlighted in bead representations. A surface representation figures out the TM domain backbones. The NRP1 TM monomers are figured in blue or cyan. The PLXA1 TM monomers are figured in red or pink. The grey surfaces represent the polar heads of the POPC. **A:** NRP1 homodimer; **B:** NRP1-PLXA1 heterodimer; **C** and **D** PLXA1 homodimer in left-handed structure (**L**) and in right handed structure (**R**) respectively.

Helix-helix contacts were evaluated by calculating the average distance matrices. The contact maps exhibit the residue pairs having the smallest distances between the backbone beads on average over a time range for which the dimer is completely stabilized. All the distance matrices corresponding to the two homodimers and the heterodimer were calculated and the resulting contact maps were compared. An overview of all these contact maps indicates that the number of residue pairs having the smallest distance between their backbone beads is greater in POPC than in DOPC. The maps in figure 3 display the major characteristics of the helix-helix interfaces of each dimer. Representative structures in the lipid bilayer are shown in figure 4 with key interfacial residues highlighted.

NRP1 homodimer contact maps are all asymmetric, suggesting that the two helices are slightly shifted along the Z axis and/or slightly rotated about their own helical axis. The variability of the helix-helix interfaces is clearly evidenced but all exhibit the same key residues that confer dimer stability, namely residues Met_12_, Gly_16_ and Gly_20_, which define the interacting motif M_12_xxxG_16_xxxG_20_. Gly_24_, the last residue of the double motif is not systematically observed at the interface and the contact distances are generally greater than 0.6 nm. Gly_24_ does not seem to be a key residue for helix-helix stability. Other residues in proximity to these key residues or aligned on the same helix face are observed at the interface, such as the Thr_8_ residue seen in the POPC bilayer.

The right-handed and the left-handed structures of the PLXA1 homodimer induce significant differences in helix-helix interfaces. The contact maps associated to right-handed packing are practically symmetric whereas those associated to left-handed packing are asymmetric. The right-handed structures exhibit well conserved interfacing residues namely Gly_11_, Gly_15_, and Leu_19_ included in the motif G_11_xxxG_15_xxxL_19_. The presence of Leu_19_ at the interface is better represented in POPC than in DOPC. The surrounding residues Ile_7_ and Gly_12_ are frequently observed at the interface. The predominant interfacing residues of the left-handed structures are Ala_6_, Gly_9_, and Gly_13_. These three key residues form the interacting motif A_6_xxG_9_xxxG_13_ both in DOPC and in POPC. Ile_10_ is also frequently observed at the helix-helix interface.

The right-handed structure of the heterodimer shows that each of the two monomers exhibits the same interfacing residues identified in the two right-handed structures of the homodimers. Contact maps are generally asymmetric but all exhibit the same pattern with the key interfacial residues Met_12_, Gly_16_ and Gly_20_ for the NRP1 TM domain and Gly_11_, Gly_15_, and Leu_19_ for the PLXA1 TM domain. Residues adjacent to the key residues are also involved in interfacial contacts. Contact distances are generally larger than 0.6 nm but their presence is almost systematic.

### Free Energy of Dissociation

**Figure 5:**
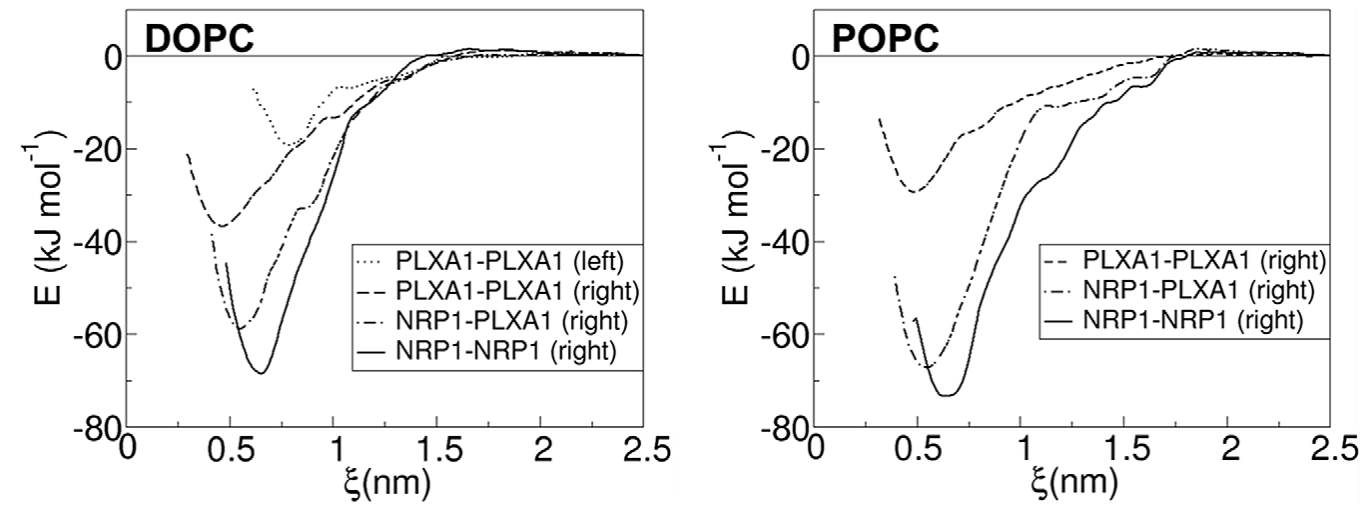
Potential of mean force evaluated by umbrella sampling calculations at a sampling of 2 µs per window in DOPC and POPC of the NRP1 and PLXA1 homodimers and the NRP1- PLXA1 heterodimer. The PMF profiles correspond to the right-handed structure of the dimers in both bilayers. The PMF profile evaluated for the left-handed structure of the PLXA1 homodimer seen in the DOPC bilayer is shown.

The free energies of dissociation of the TM domains were evaluated in the DOPC and POPC environments. For the two milieus, the PMF profiles were calculated by considering the right-handed structure of the homo and hetero dimers. In addition, the free energy of dissociation of the PLXA1 TM domains packed in a left-handed manner was evaluated only in DOPC where this packing mode is well represented (see Table 2). The very weak representation of this structure in POPC suggests a very unstable dimer making this state not pertinent for PMF calculations. The results are summarized in Table 3 and the PMF profiles are shown in figure 5.

**Table 3:** Characteristics of the PMF profiles calculated for the dimers in DOPC and POPC bilayers.

Dimer		DOPC		POPC		DOPC	POPC
		E_min_	D_min_	E_min_	D_min_	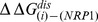	
PLXA1homodimer	R	−36.6 (1.1)	0.47	−29.3 (0.9)	0.49	28.9	44.4
	L	−19.4 (0.8)	0.79			47.7	
NRP1homodimer	R	−68.7 (1.4)	0.65	−73.4 (2.2)	0.64	0	0
NRP1-PLXA1heterodimer	R	−59.2 (1.7)	0.56	−67.3 (1.4)	0.55	8.0	5.1

The PMFs correspond to the separation of the monomers associated in right-handed interactions (R). The PMF profile was calculated in the case of the separation of the PLXA1 monomers associated in left-handed (L) within a DOPC bilayer. E_min_ is the difference in free energy between the associated state and the full separation of the monomers. D_min_ is the distance separating the center of mass of the backbone beads of the two helices at the minimum of the PMF profile. 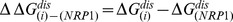 is the difference between the apparent dissociation free energy of the PLXA1 homodimer or the heterodimer (i) and the apparent dissociation free energy of the NRP1 homodimer (see equation 2). Energies are given in kJ mol^−1^ and distances are in nm.

PMF profiles calculated in DOPC show that the lowest global minimum is obtained for the NRP1 homodimer with a free energy of dissociation estimated at −68.7 kJ mol^−1^. Compared to the values evaluated for the right-handed structures of the heterodimer and the PLXA1 homodimer, differences in free energies are about 10 kJ mol^−1^ and 32 kJ mol^−1^ respectively. Therefore, the separation of the two PLXA1 monomers crossed in right-handed interactions appears relatively easy compared to the two other dimers. However, the separation of these monomers crossed in left-handed interactions is even easier. The very large difference in free energy of dissociation of 49 kJ mol^−1^, relative to the lowest minimum, evidences a very weak power of association in a left-handed mode. We note that these differences in free energies calculated from equation (2) are in the same order of magnitude.

PMF profiles differ by the deepness of the global minima but also by their position. The minimum is at 0.47 nm for the right-handed PLXA1 homodimer, which corresponds to the shortest inter-helix distance compared to 0.56 nm for the heterodimer and 0.65 nm for the NRP1 homodimer. The position of the minimum for the left-handed structure of the PLXA1 homodimer corresponds to the largest inter-helix distance, 0.32 nm larger than for the right-handed mode. As a result, the distance of approach of the monomers is not correlated to the strength of the interactions. In both cases the free energy increases more or less rapidly with different regimes during the separation of the monomers but without energy barriers. The full separated state is reached at ∼1.6 nm for all the dimers.

PMFs calculated in the POPC environment exhibit the same features as those observed in DOPC (figure 5). The deepest global minimum is observed for the NRP1 homodimer (−73.4 kJ mol^−1^) very close to the deep minimum observed for the heterodimer. The difference in free energies is around ∼6 kJ mol^−1^. More remarkable is the very weak free energy of dissociation of the two PLXA1 TM domains evaluated at −29.3 kJ mol^−1^ which means a destabilization of ∼44 kJ mol^−1^ and 38 kJ mol^−1^ compared to the NRP1 homodimer and the heterodimer respectively. Here again, the scale of the free energies is in agreement with that calculated from equation (2). As observed previously, PMF profiles exhibit several regimes over the course of the separation of the monomers without energy barriers. The positions of the global minima are practically identical to those observed in DOPC, albeit slightly shifted towards larger inter-helix distances, at the most 0.02 nm. The shortest inter-helix distance is observed for the PLXA1 homodimer. The full separated states occur at inter-helix distances of 1.8 nm for the NRP1 homodimer and the heterodimer and 1.6 nm for the PLXA1 homodimer.

PMFs calculations demonstrate the effect of the environment on the dissociation of the monomers. This effect is weak for the NRP1 homodimer and the heterodimer. Free energies of dissociation are slightly greater in POPC than in DOPC with a gain of −4.7 kJ mol^−1^ and −8.1 kJ mol^−1^ respectively. These values indicate a weak stabilization effect of the assembly of the TM domains. Lipid effects are more clearly evidenced for the PLXA1 homodimer. This dimer is unstable in the two milieus, but this instability is intensified in POPC. The differences in the apparent dissociation free energies ΔΔG^dis^ reveal an increase in destabilization of the PLXA1 homodimer of over 15 kJ mol^−1^ in POPC with respect to the NRP1 homodimer.

These calculations evidence the effect of the lipids on the dimerization power of TM domains. They clearly demonstrate the weakness for self-association of the PLXA1 TM domains both in right-handed and in left-handed interactions. This finding could suggest the possibility of equilibrium between the two forms of packing.

Thus, the PMF profiles calculated for the homodimers and the heterodimer embedded within a DOPC and a POPC bilayer reveal the existence of a hierarchy of interaction strengths between the TM domains. The results indicate that the two NRP1 TM domains exhibit a very strong power of association, slightly stronger than that of the NRP1-PLXA1 TM domains. Conversely, the strength of association of the two PLXA1 TM domains is considerably lower. The scale of interaction energies of the intramembrane domains can be summarized as follows: NRP1-NRP1≥ NRP1-PLXA1>>PLXA1-PLXA1, a hierarchy in agreement with the results of the BACTH two-hybrid assay.

## Discussion

This work focuses on the understanding of the Sema3 receptor complexes involving plexins A and neuropilins. The multiple functions of these secreted semaphorins (Sema3s) depend on signaling through class A plexins. Alone, plexins cannot function as semaphorin receptors and neuropilins are required as co-receptors. Consequently, for signaling through plexin A, class 3 Semaphorins have to interact with neuropilins that in turn have to interact with plexins.

In this paper, we have addressed the contribution of the transmembrane domains of neuropilin 1 and plexin A1 in receptor association using CG-MD simulations in lipid bilayers. Our focus on TM interactions comes from the intriguing presence of single or double GxxxG motifs in almost all known neuropilin partners. Here our observations showing that the PLXA1 TM sequence is also able to dimerize with NRP1 TM suggest that blocking the interaction of NRP1 TM with PLXA1 TM may also be an efficient therapeutic strategy. The simulations presented here provide a unique source of information contributing to the understanding of the action mechanism of such peptidic antagonists. They demonstrate the existence of dynamic TM-TM interactions which determine the hierarchy of the receptor complex formation.

### CG-MD Simulations Predict a Hierarchy of TM Interactions

CG-MD simulations in DOPC and POPC bilayers demonstrated that the NRP1 and PLXA1 TM domains spontaneously assemble to form homodimers and heterodimers. Our results point to two major findings. One is the very high propensity for a right-handed packing of the helices and the second is the low propensity for a left-handed packing mode when at least one PLXA1 TM monomer is involved in association (Figure S1).

We show that the PLXA1 homodimer is allowed to visit the two binding modes at a significant level in the two lipid environments, contrarily to the heterodimer for which the left-handed association is observed at low level. The propensity of the TM helices to associate in right or left handed manner is modulated by the nature of the environment. Also, the strength of helix-helix interactions differ in the two milieus as deduced from PMF calculations. Similar effects of lipids on the self-assembly of peptides have been reported in several other CG-MD studies [52]–[54]. We have to note here that only one starting structure was used to evaluate these effects. In addition, because of the high computational cost, we did not test the dependence on starting conditions nor the sensitivity of the PMFs on the initial configurations used in each window.

The differences in energy minima of the PMF profiles calculated in DOPC and POPC are around 8 kJ mol^−1^ or less as evaluated for the NRP1 homodimer. Lipids effects on the power of TM domain association exist but are weak. According to various studies, these differences in energies may not be significant because of statistical and systematic errors that could influence free energies [52], [55].

Intriguing is the large destabilization of the right-handed binding mode of the PLXA1 homodimer in POPC (more than 15 kJ mol^−1^) with respect to the more stable mode of the NRP1 homodimer. This result may suggest that the PLXA1 TM domains are more sensitive to the nature of the environment during the association than the two other dimers. We also remark that there is no relation between the preferred binding mode and the strength of TM interactions. Left-handed interactions are more often observed in DOPC while right-handed interactions are more often observed in POPC, but despite this preference of binding mode, the strength of the interactions is weaker than in DOPC. We speculate that these results are both related to the differences in the sequence of the TMs and to the nature of the lipids. An effect of the bilayer thickness and its fluidity can be hypothesized. In this study we did not focus on the role of the lipids in modulating association of TM domains.

The left-handed packing of the PLXA1 homodimer leads to the weakest free energy of dissociation as estimated from PMF calculations, revealing a very unstable dimerization state. Weak interactions facilitate the complete dissociation of the monomers, as seen in POPC. Within the bilayer the monomers can move separately for more than 5 µs before they re-associate in right-handed interactions for the rest of the simulation. This behavior reveals the existence of a dynamic equilibrium between monomers and dimers. Monomer-dimer equilibrium has been reported in CG simulations of the GpA dimer [56]. In addition, the alternate succession of helices crossed with a positive and a negative angle (or inversely) without dissociation demonstrated the existence of a rapid exchange between the two modes of association.

Association/dissociation events have been also described in the literature, such as for example on WALP peptides and GpA wild and mutant peptides [55], [57]–[59]. Together, the existence of a bimodal packing of the PLXA1 TM helices and of an equilibrium between monomers and dimers suggests the possibility of dynamic exchanges between several other intramembrane domains of the various plexin partners including neuropilins. This is consistent with the demonstration that a peptide mimicking the TM of PlexA1 is as efficient as a peptide mimicking the TM of NRP1 to perturb receptor assembly and subsequent signaling pathway.

An important finding is the possibility of left-handed packing when both the NRP1 and PLXA1 TM domains are embedded in DOPC or in POPC bilayers. As evidenced, these interactions are less frequent than for the PLXA1 homodimer but, similarly to what was found for this homodimer, DOPC seems to favor this type of interaction.

Contrarily to these two dimers, NRP1-NRP1 TMs were never detected in a left-handed binding mode, neither in DOPC (Figure S1) nor in POPC (data not shown) during a total of 200 µs simulations. A unique mode of binding is evidenced. The presence of the double GxxxG motif in the TM sequence is in total coherence with this finding. However, taking in mind the problem of conformational sampling and the lack of binding/unbinding events within the time scale simulated, we cannot exclude other potentially relevant binding modes. Alternative approaches such as 2D PMF along the distance and rotation coordinates could be applied for the search of other binding modes [60]. In the same line, we have performed analysis of the 1D PMF runs in the two environments along the distance coordinates. No other binding mode than the one described from our CG-MD simulations was distinguished. Once the two helices are far apart, helix-helix reorganization was not observed (Figure S2).

Thus, the present results tend to demonstrate that the PLXA1 TM domain encodes the properties for both left-handed and right-handed binding mode and the NRP1 TM domain encodes the properties for right-handed binding mode.

PMF analysis gives only approximate magnitudes on the strength of TM association but makes it possible to predict a hierarchy of self-association for the TM monomers. We estimate that the NRP1 TM domain displays a very strong power for homo dimerization and hetero dimerization with the PLXA1 TM domain. The strong power of interactions involving at least one NRP1 TM domain reflects specific interactions which could drive dimerization of the full length protein. Neuropilin 1 dimers preexist at the cell surface in the absence of ligand before the formation of complexes [26], [45]. Contrarily, the PLXA1 TM domain displays a very weak power for homo association and this could be the result of unspecific interactions, explaining the modulator role of this receptor in the formation of neuropilin 1 receptor complexes.

### GxxxG Motifs Drive TM Interactions

Dimer structures are well defined and the same interacting helix faces were identified in DOPC and POPC bilayers. Each helical TM exhibits its own face for dimerization and we demonstrate that the TM sequences encode an intrinsic propensity to self-associate in the presence of the GxxxG motifs. For NRP1 the key interacting residues constitute the M_12_xxxG_16_xxxG_20_ motif as a guide for homo and hetero dimerization. The C-terminal Gly_24_ of the double motif (G_16_xxxG_20_xxxG_24_) is not involved in tight packing albeit present at the interface. The Gly rich sequence G_9_IGGGGG_15_ of PLXA1 is also involved both in homo and hetero dimerization and, together with surrounding residues, gives rise to two distinct association properties. With the small Ala_6_ residue at the N-terminus, the A_6_xxG_9_xxxG_13_ motif confers the specificity of left-handed interactions for the homodimer. With Leu_19_ at the C-terminus, the G_11_xxxG_15_xxxL_19_ motif confers the specificity of right-handed interactions for the homodimer and the heterodimer.

The role of the sequence context in stabilizing helix–helix interactions mediated by a GxxxG motif is here very well illustrated. According to the general principles for transmembrane stability, the GxxxG motif is necessary but not sufficient to achieve strong TM helix–helix association and the sequence context modulates the stability of the TM helix-helix packing [61].

In the case of NRP1, Met_12_ is aligned on the same helix face as G_16_, G_20_ and G_24_ and with the flanking Ser residue contributes to dimer stabilization through polar and Van der Waals interactions. In the case of PLXA1, Ala_6_ is a weak stabilizing residue, but with Ile_7_ it contributes to Van der Waals stabilization of the left-handed structure. Leu_19,_ with the proximal residues, contributes to Van der Waals stabilization of the right-handed structure. These are the factors identified as involved in controlling the stability of a GxxxG stabilized TM helix dimer [8].

### “Versatile” PLXA1 TM Domain

**Figure 6:**
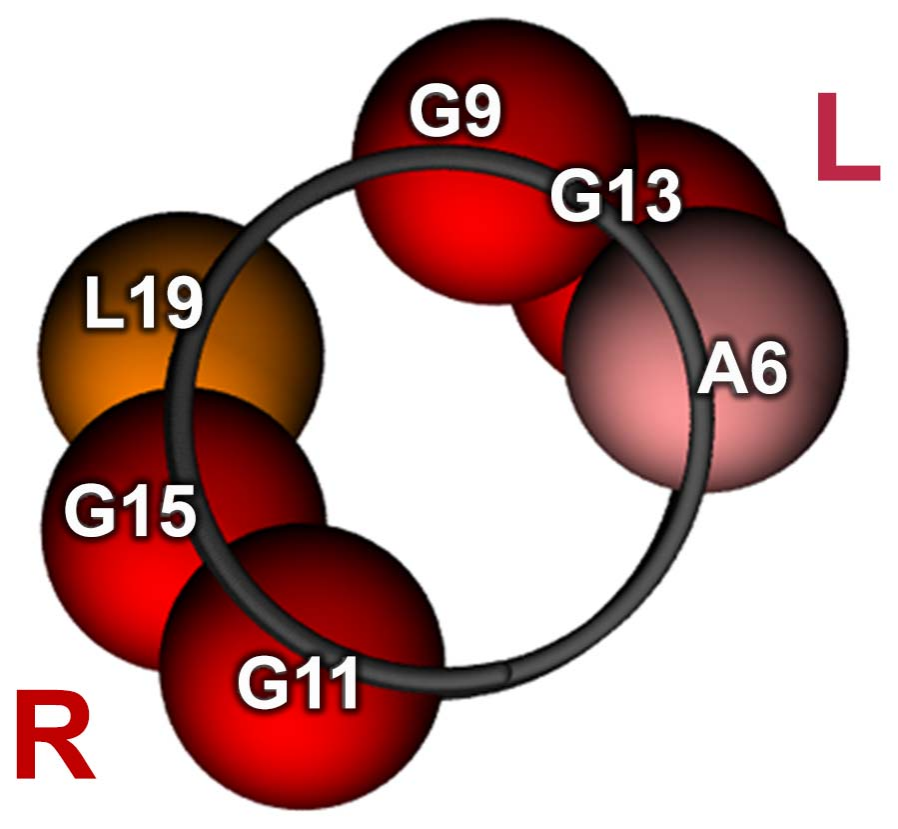
Position of the key residues involved in PLXA1 TM homodimerization. **R** denotes the helix face conferring a right-handed packing and **L** denotes the helix face conferring a left-handed packing.

Interacting key residues responsible for the specificity of left-handed and right-handed interactions of the PLXA1 TM homodimer are distributed on the two opposed faces of the intramembrane helix. This major finding is clearly illustrated in figure 6. This result is of considerable importance because the two helix faces can serve for distinct interactions leading to a large diversity of partners. These properties are very probably useful in explaining the versatile character of semaphorin - neuropilin - plexin complexes known to be regulators of tumor progression and tumor angiogenesis [22]. The dynamic equilibrium between the two modes of helix packing governed by the presence of distinct motifs could induce the possibility of switching from one mode of packing to another one depending on the partner. This hypothesis is supported by a similar behavior reported for the ErbB receptors [62]–[65].

Within the TM domain of most human ErbB receptor tyrosine kinases, two GxxxG like motifs are conserved and interactions mediated by one motif guide the receptor in active state, whereas interactions mediated by the second motif guide the receptor in inactive state. Thus, the ErbB receptor function could depend on the TM dimer alternating in a switch-like fashion between two structures, stabilized by either of the two GxxxG-like motifs. Moreover, some residues involved in ErbB2 homodimerization are also involved in ErbB1/ErbB2 heterodimer formation. The core of dimerization is conserved and the surrounding residues which contribute to the stability of the structures of the ErbB2 homodimer or the ErbB1/ErbB2 heterodimer differ [66]. Thus, the properties of the ErbB TM domains parallel those described here for the PLXA1 TM domain. The core of dimerization appears to be the G_11_G_12_G_13_ sequence common to the two motifs A_6_xxG_9_xxxG_13_ and G_11_xxxG_15_xxxL_19_ which allows rapid switching between the two modes of association. The first motif is responsible for homodimerization and guides left-handed interactions and the second motif is responsible for homodimerization and heterodimerization and guides right-handed interactions.

## Conclusion

### Toward the Design of Therapeutic Agents

As a conclusion, CG-MD simulations enable the description of models for TM interactions of Sema3 co-receptors. To the best of our knowledge, this work is the first devoted to investigations on the stability and specificity of TM interactions of the Sema3 co-receptor in membrane bilayers.

The success of coarse-grained models has been proven in many studies on membrane protein systems [67] and thus gives us confidence in the biological relevance of our results. Ongoing research aims at exploring these complex systems in more sophisticated bilayers including mixed lipids and cholesterol to corroborate our models.

The GxxxG motifs play critical roles in transmembrane association and it can be expected that TM interactions will influence the association of full-length Sema3 receptors. The present data agree with the first experimental studies on neuropilins 1 [21], [45], [46] and open the route for additional studies to explore experimentally the role of the TM domains of class A Plexins and neuropilin members that can now be extended to receptor tyrosine kinases to have a full picture of the Semaphorin signaling platform [68].

Many types of tumor cells express semaphorin receptors. These transmembrane receptors represent major potential advances toward the design of a new class of efficient therapeutic agents for cancer treatment [10], [21]. The therapeutic potential of the NRP1 TM domain has been already demonstrated [45], [46] and we anticipate that the properties of association of the intramembrane domains of plexin A1 and neuropilin 1 reported here will contribute to therapeutic perspectives. The computational rational design of hydrophobic peptides able to specifically bind to TM domains of receptors, and alter their functions, has already been successfully employed in the case of integrins [69]. Our present work should pave the way for the design of such interfering peptides, defining a novel class of therapeutic agents.

## Materials and Methods

### Estimation of TM Domains Dimerization Propensity (BACTH Method)

A bacterial two-hybrid system based on the recombination of adenylate cyclase CyaA from *Bordetella pertussis* was used to measure both homo- and heterodimerization propensities of transmembrane domains of interest. This system relies on reconstitution of the catalytic domain of CyaA which can be separated into two complementary fragments [70]. When each fragment is fused to a protein of interest, a functional adenylate cyclase can be reassembled upon interaction of the two proteins, which is followed by the production of cyclic AMP in an *E. coli* strain lacking its own adenylate cyclase. The synthesis of cyclic AMP is proportional to the association propensity of the two fragments, and can be measured in a number of ways [70]. This system has been used widely and is readily amenable for study of membrane proteins interactions. Very briefly, we have modified the BACTH pKTN25 and pUT18 plasmids so that they encode for hybrid proteins containing an OmpA signal sequence followed by the different TM domains of interest and the T25 and T18 fragments of adenylate cyclase (Sawma et al., manuscript submitted). The following transmembrane sequences were used: ILITIIAMSALGVLLGAVCGVVLYRKR for NRP1, LLTLPAIVGIGGGGGLLLLVIVAVLYAYRRK for PLXA1 and IEITLIIFGVMAGVIGTILLISYGIRRK for the GpA control. These sequences contain a C-terminal tribasic sequence to ensure their correct insertion, as was previously done for studies with synthetic peptides [45], [46]. Double transformation in BTH101 (cya^-^) *E. coli* cells, cell growth and induction, colorimetric assay on MacConkey reporter plates and beta-Galactosidase assay in 96-well arrays on a TECAN machine were performed as described [70].

### Molecular Dynamics System Setup

CG-MD simulations were performed to investigate the homo and hetero association of NRP1 and PLXA1 TM domains using the GROMACS software package (version 4.5.4) [71], [72]. The TM peptides, the lipids and the water CG particles were described with the MARTINI force-field version 2.1 [49], [50] extensively used for the study of various peptide-lipid membrane systems [54]. Images were created using the VMD software [73] (http://www.ks.uiuc.edu/Research/vmd/).

### TM Peptide Sequences

The two TM sequences used in the simulations correspond to the native potential sequences as found in the Uniprot database (http://www.uniprot.org/) (Table 1). We adjusted the length of the TM domains by adding a few residues on each side, in order to design a 28 amino acid sequence. For the sake of clarity, the TM sequences have been re-numbered 1 to 28 throughout this paper.

The TM domain of NRP1 is characterized by two successive GxxxG motifs G_16_xxxG_20_xxxG_24_. The PLXA1 TM sequence is characterized by the presence of 6 Gly residues, 5 being consecutive in the sequence constituting the G_11_GGGG_15_ motif. Another GxxxG like motif, G_9_IGGG_13_, is observed overlapping the previous one. Considering the α helical structure of the TM domains, the 6 Gly residues of the PLXA1 TM domain are distributed over all the helix faces, covering almost two helix turns, while the 3 Gly residues in the NRP1 TM domain are aligned on only one helix face.

### Bilayer Preparation

We prepared large CG DOPC (1,2-di-oleoyl-*sn*-glycero-3-phosphocholine) and POPC (palmitoyl-2-oleoyl-*sn*-glycero-3-phosphocholine) bilayers for TM domain self-assembly studies. The pre-equilibrated DOPC bilayer taken from the MARTINI web site (http://md.chem.rug.nl/cgmartini/) was replicated onto a 2×2 grid in the bilayer plane (X, Y directions). The POPC bilayer was built from the original pre-equilibrated POPE bilayer. The POPE bilayer was replicated onto a 2×2 grid in the bilayer plane as for the DOPC bilayer. After minimization, 1 µs of equilibration was performed using the conditions given in the following. The POPC bilayer was obtained by replacing the first CG particle (Qd NH3 in POPE was replaced by Qo NC3 in POPC).

A pure DOPC bilayer containing 492 lipids and 6000 water particles, and a pure POPC bilayer composed of 451 lipids and 5844 water particles were obtained. After minimization, each bilayer was equilibrated for 1 µs using the following conditions. The temperature was coupled to a thermostat at 300 K using a Berendsen algorithm [74] with a time constant of 1 ps applied separately to the lipids and the water groups. The pressure was coupled using a semi-isotropic scheme (coupling time 0.5 ps, compressibility 4.5×10^−5^ bar^−1^) in which the lateral and perpendicular pressures are coupled independently at 1 bar. A time step of 0.025 ps was used. After equilibration the X, Y, Z dimensions of the bilayer were 13.61, 12.1, 8.73 nm and 11.96, 11.94, 9.22 nm for the DOPC bilayer and the POPC bilayer respectively. The Z axis represents the normal to the bilayer plane (X, Y).

### System Preparation

The CG structures of the NRP1 and the PLXA1 TM domains were modeled as ideal α helices from the atomistic structure initially built using AMBER [75], [76]. The C-terminus was capped with a negative charge, and the N-terminus was capped with a positive charge as given in the MARTINI force field. The aspartic acid residue included in the NRP1 TM sequence was considered to be uncharged. Thus, the two TM domains were electrically neutral. Both the NRP1 and PLXA1 monomers were aligned along the helical axis before applying the insertion procedure.

Two monomeric peptides were inserted in a parallel manner in the equilibrated bilayer (DOPC/POPC) at a distance of 6 nm from each other. Prior to insertion, the TM peptides were aligned along the normal to the bilayer and were randomly rotated about the helix axis. This leads to a random orientation of GxxxG motifs of one monomer relative to the second. The resulting initial helix-helix interface is thus totally arbitrary. A small number of lipids and water molecules (a few tens depending on the systems) were removed from the sites of peptide insertion.

The system was energy minimized for 10000 steps using the steep integrator without peptide particle restraint. MD simulations were then performed at 300 K with a coupling time of 1 ps applied separately to the protein, the lipids (DOPC/POPC) and the water groups using a v-rescale algorithm. The pressure was coupled using a semi-isotropic scheme, in which the lateral and perpendicular pressures were coupled independently at 1 bar with a coupling time of 5 ps and a compressibility of 3×10^−4^ bar^−1^. A time step of 0.040 ps was used for the production step. For all the simulations (pure bilayers and peptide-lipid bilayers) Lennard Jones and Coulombic interactions were shifted to zero between 0.9 and 1.2 nm and 0.0 and 1.2 nm respectively.

Six systems were studied. Three systems contained two peptides embedded in a DOPC bilayer and three systems contained the same two peptides embedded in a POPC bilayer, the two peptides being identical (homodimer) or different (heterodimer). The simulations were repeated several times, varying the initial orientation of the two monomers relative to each other. Four distinct simulations were performed for self-association studies of the NRP1 TM domains, three in the case of the PLXA1 TM domains, and six in the case of the heterodimer, for each type of bilayer. Simulations of 15 µs or longer were performed depending upon the dimer type, but sufficiently long to observe the assembly of the two peptides and the stability of association for at least 10 µs. A total of 13 simulations were performed in each lipid bilayer, resulting in a total simulation time of 613 µs (Table 2).

### System Analysis

Tools provided in the Gromacs package were used for the analyses. The associated state of the dimer was defined from the analysis of inter-helix distance (distance between the center of mass of the helix backbones, COM) and of inter-helix contact distance (minimal distance between the helix backbones). The two helices were considered associated when the closest contact distance was less than 0.5 nm.

The RMSD (Root Mean Square Deviation) matrix, which compares all the conformations generated during the simulation, was calculated to identify the periods of the simulation during which the dimer structure is stabilized. A dimer is stable when the RMSD value (calculated over the backbone beads) is less than 0.3 nm. The longest period of the dimer stabilization was chosen to calculate the contact matrix that represents the smallest distances between the backbone beads of the residue pairs, on average. The helix-helix interface is then extracted from this contact matrix.

For the NRP1 homodimer and the NRP1-PLXA1 heterodimer, the simulations led generally to only one major state for the association of the helices. For the PLXA1 homodimer two distinct states were observed along each of the six simulations. As detailed in the following, the two helices were successively crossed with a positive or a negative crossing angle (see results). Then, for the analysis, the trajectory portions corresponding to the positive mode of association were merged and similarly, the trajectory portions corresponding to the negative mode of association were merged.

### PMF *via* Umbrella Sampling

To compare the strength of interaction of the NRP1 homodimer, the PLXA1 homodimer and the NRP1-PLXA1 heterodimer, we calculated the potential of mean force (PMF) following the procedure described by Marrink and coll [54].

PMFs were calculated by carrying out a series of umbrella sampling simulations, before unbiasing with the weighted histogram analysis method (WHAM) [77]. To conduct umbrella sampling, we generated a series of configurations along the reaction coordinate, ζ, chosen as the distance between the centers of mass of the backbone beads of the two helices. A pulling simulation was performed over the course of 420 ns in the X and Y dimensions (membrane plane). Starting from two associated helices, taken from the self-assembly simulations, the two helices were dissociated by restraining the backbone beads of one helix with a force constant of 1000 kJ mol^−1^ nm^−2^ and applying constant velocity pulling (pull rate = 0.01 nm.ps^−1^). For each system, 22 configurations, corresponding to a 0.1 nm shift of the monomer, were chosen as starting configurations for the umbrella sampling windows that were run independently. Each configuration was equilibrated for 200 ns followed by 2 µs production. The distances between the centers of mass of the backbone beads of the two helices were sampled from 0.5 nm to 2.6 nm for the NRP1 homodimer and the heterodimer, and from 0.4 nm to 2.5 nm for the PLXA1 homodimer. Seven PMF profiles were calculated corresponding to 310 µs of total simulation time.

PMF extraction using WHAM yielded the ΔG for the binding/unbinding process. By integrating the PMF profile, we calculated the association constant K_A_ which is defined in cylindrical coordinates by the following expression [52], [54].
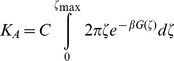
(1)ζ_max,_ represents the cylindrical radius separating the associated and dissociated states of the two TM helices defined to be 2.5 nm. C = 1 for the heterodimer and C = 1/2 for the two homodimers. From the association constant, we determine the apparent dissociation free energy given by ΔG^dis^ =  −RTlnK_A_. Since a direct comparison between the calculated value and quantitative experimental data is difficult, we determined ΔΔG^dis^ given by the following expression:
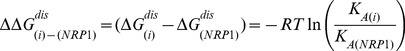
(2)With RT = 2.4943 kJ mol^−1^


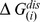
 is the apparent dissociation free energy of the PLAX1 homodimer or the NRP1-PLAX1 heterodimer.


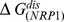
 is the apparent dissociation free energy of the NRP1 homodimer taken as reference.

## Supporting Information

Figure S1 **Crossing angle as a function of simulation time.** Time evolution of the crossing angle of the helices for the three dimers NRP1-NRP1, NRP1-PLXA1, PLXA1-PLXA1 in DOPC. Several independent CG-MD simulations were performed for each dimer. In the case of NRP1-NRP1 only the right-handed binding mode is observed (negative value of the crossing angle). The multiple independent simulations arrived at the same dimer structure. The right-handed binding mode is preferred for the heterodimer, but left-handed transitions are observed. The PLXA1-PLXA1 homodimer evidences frequent right and left-handed transitions.
(TIF)

Figure S2 **The motions of one helix relatively to the other.** Analysis of the 1D PMF runs for the dimers NRP1-NRP1, NRP1-PLXA1 in the right-handed binding mode and PLXA1-PLXA1 in right and left-handed binding modes. The graphs represent the X Y coordinates of the center of mass of each helix during the Umbrella Sampling calculations in DOPC (22 windows). Helix H1 is restrained at the center of the lipid box and H2 is allowed to move in the XY plane for each value of the reaction coordinate ζ. The NRP1-NRP1 and NRP1-PLXA1 dimers dissociate without reorganization. For the right-handed PLXA1 homodimer, a reorganization of the helices is suggested at the first steps of the dissociation process.
(TIF)
