# Supplementary figures and images for: Transmembrane Recognition of the Semaphorin Co-Receptors Neuropilin 1 and Plexin A1: Coarse-Grained Simulations
Source: PLoS One. 2014 May 23;9(5):e97779. doi: 10.1371/journal.pone.0097779 (PMC4032258; doi:10.1371/journal.pone.0097779)

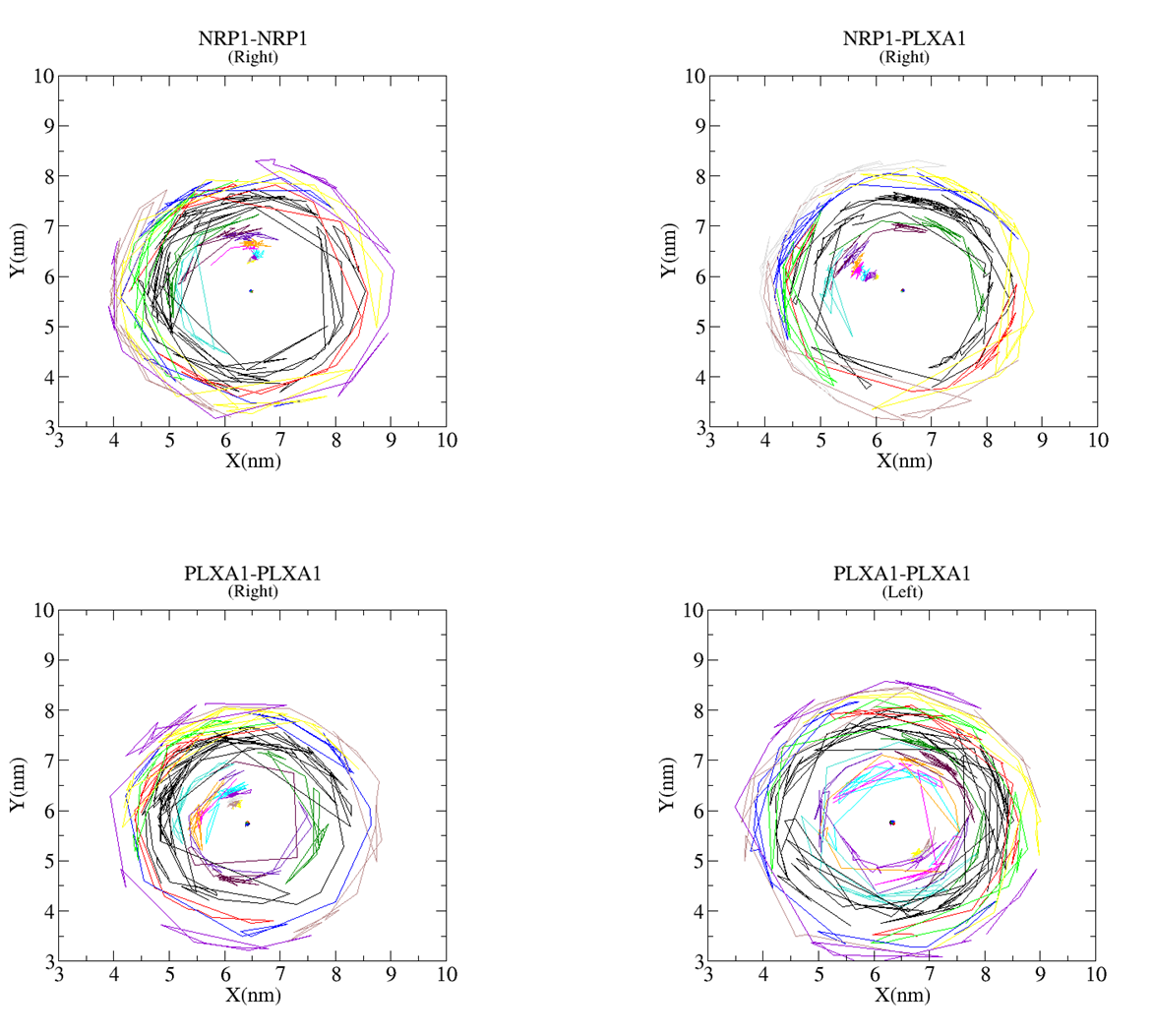

Supplement: Figure S2 — The motions of one helix relatively to the other. Analysis of the 1D PMF runs for the dimers NRP1-NRP1, NRP1-PLXA1 in the right-handed binding mode and PLXA1-PLXA1 in right and left-handed binding modes. The graphs represent the X Y coordinates of the center of mass of each helix during the Umbrella Sampling calculations in DOPC (22 windows). Helix H1 is restrained at the center of the lipid box and H2 is allowed to move in the XY plane for each value of the reaction coordinate ζ. The NRP1-NRP1 and NRP1-PLXA1 dimers dissociate without reorganization. For the right-handed PLXA1 homodimer, a reorganization of the helices is suggested at the first steps of the dissociation process. (TIF) [file pone.0097779.s002.tif]
